# Mutation of *pescadillo* Disrupts Oligodendrocyte Formation in Zebrafish

**DOI:** 10.1371/journal.pone.0032317

**Published:** 2012-02-23

**Authors:** Timothy Simmons, Bruce Appel

**Affiliations:** 1 Department of Biological Sciences, Vanderbilt University, Nashville, Tennessee, United States of America; 2 Department of Pediatrics, University of Colorado Anschutz Medical Campus, Aurora, Colorado, United States of America; National Institutes of Health/NICHD, United States of America

## Abstract

**Background:**

In vertebrates, the myelin sheath is essential for efficient propagation of action potentials along the axon shaft. Oligodendrocytes are the cells of the central nervous system that create myelin sheaths. During embryogenesis, ventral neural tube precursors give rise to oligodendrocyte progenitor cells, which divide and migrate throughout the central nervous system. This study aimed to investigate mechanisms that regulate oligodendrocyte progenitor cell formation.

**Methodology/Principal Findings:**

By conducting a mutagenesis screen in transgenic zebrafish, we identified a mutation, designated *vu166*, by an apparent reduction in the number of oligodendrocyte progenitor cells in the dorsal spinal cord. We subsequently determined that *vu166* is an allele of *pescadillo*, a gene known to play a role in ribosome biogenesis and cell proliferation. We found that *pescadillo* function is required for both the proper number of oligodendrocyte progenitors to form, by regulating cell cycle progression, and for normal levels of myelin gene expression.

**Conclusions/Significance:**

Our data provide evidence that neural precursors require *pes* function to progress through the cell cycle and produce oligodendrocyte progenitor cells and for oligodendrocyte differentiation.

## Introduction

In the vertebrate nervous system, the rapid and efficient transmission of electrical impulses along many axons requires the presence of an insulating myelin sheath. In the central nervous system (CNS), the myelin sheath is formed by a population of glial cells known as oligodendrocytes [1]. Within the spinal cord, most oligodendrocytes arise from a ventral region known as the pMN domain, which also gives rise to motor neurons and interneurons [2]. Once specified, oligodendrocyte progenitor cells (OPCs) migrate out of the pMN domain toward their target axons in the lateral spinal cord. These OPCs divide and extend multiple fine membrane processes as they migrate [3]. Upon reaching their target axons, OPCs stop dividing and extend processes that contact and wrap multiple axons in tube-like structures that are then compacted to form the myelin sheath [4].

The mechanisms that pattern the dorsoventral axis of the neural tube to establish pMN precursors are thoroughly described [5]. By contrast, the mechanisms that specify OPCs from pMN precursors and regulate their subsequent division, migration and differentiation to myelinating cells are poorly understood. In an effort to fill this gap we conducted an ENU based mutagenesis screen in zebrafish carrying the *Tg(olig2:EGFP*) reporter, which labels pMN precursors and oligodendrocyte lineage cells [6]. Here we describe one mutation, *vu166*, which causes a reduction of oligodendrocytes. We determined that the *vu166* allele is a nonsense mutation of *pescadillo* (*pes*), which has been implicated in ribosome biogenesis. Our analysis indicates that the oligodendrocyte deficit of *pes* mutant larvae results from disruption of cell cycle progression among neural precursors. In addition, we found that OPCs fail to migrate normally in mutant embryos as a consequence of an altered cellular environment and that oligodendrocytes express abnormally low levels of myelin genes in mutant larvae, raising the possibility that Pes function is necessary for oligodendrocyte differentiation.

## Results

### The *vu166* mutation truncates the protein coding sequence of the *pescadillo* gene

**Figure 1:**
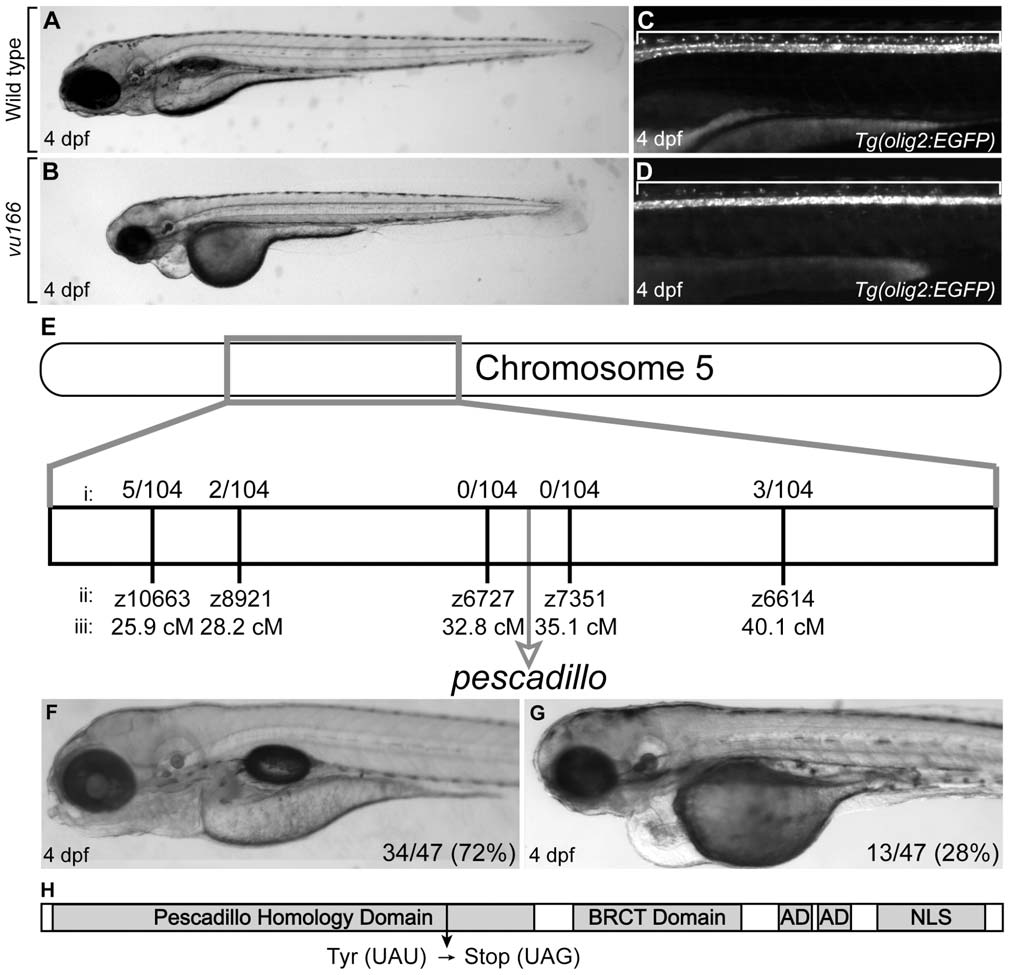
Characterization and genetic mapping of the *vu166* mutant allele. Lateral views of 4 dpf wild-type (A,C) and *vu166^−/−^* larvae (B,D). EGFP expression revealed oligodendrocytes in dorsal spinal cord of wild-type *Tg(olig2:EGFP)* larva (C, bracket). Fewer dorsal EGFP^+^ cells were evident in a *vu166^−/−^* larva (D). Bulked segregant analysis revealed that the *vu166* allele links to chromosome 5 (E). The recombination frequencies (i) at various microsatellite markers (ii) at known locations (iii) along chromosome 5 show that the mutation lies near the *pescadillo (pes)* gene locus. The *vu166* allele failed to complement the previously characterized *pes^hi2^* mutant allele, producing phenotypically wild-type (F) and mutant (G) larvae in a 3∶1 ratio. The Pes gene product (H) contains a highly conserved Pescadillo Homology domain, a BRCA1 C-terminal domain, two Acidic Domains (AD), and a Nuclear Localization Sequence (NLS). Sequencing of *pes* cDNA obtained from *vu166* mutants revealed a single base pair mutation that creates a premature stop codon predicted to prematurely truncate the protein.

We recently conducted an ethyl nitrosourea (ENU) mutagenesis screen using a *Tg(olig2:EGFP)* transgenic line of zebrafish, which expresses enhanced green fluorescent protein (EGFP) under the control of *olig2* regulatory DNA [6]. This transgenic reporter reveals dorsally migrating OPCs in living embryos (**Figure 1C**), permitting identification of mutations that disrupt the number and distribution of OPCs. We identified the *vu166* allele because homozygous mutant larvae had a deficit of dorsal spinal cord OPCs and oligodendrocytes at 4 days post fertilization (dpf) (**Figure 1D**). *vu166* mutant larvae also had defects in forebrain, eye, jaw, otolith, pectoral fin, and body length (**Figure 1B**). We used bulked segregant analysis [7] to map the *vu166* locus to chromosome 5 (data not shown). Subsequent mapping experiments revealed that the *vu166* mutation was between microsatellite markers z6727 and z7351 (**Figure 1E**). We noted that the *pescadillo (pes)* gene maps to the same genomic location [8]. A previously described mutant allele of *pes, pes^hi2^*, had a retroviral insertion in the first exon upstream of the translational start site, which abolished transcript accumulation [8], [9]. *pes^hi2^* and *vu166* mutant larvae had indistinguishable morphological defects, therefore, we performed a complementation test using *pes^hi2+/−^* and *vu166^+/−^* carriers. Approximately one quarter of the progeny had morphological defects similar to *pes* and *vu166* mutant larvae (**Figure 1F,G**) indicating that the *vu166* allele disrupts *pes* function. Sequencing of *pescadillo* genomic DNA obtained from *vu166* mutant larvae revealed a single base pair change predicted to introduce a premature stop codon in the Pescadillo homology domain of the protein (**Figure 1H**). Therefore, we hereafter refer to the *vu166* allele as *pes^vu166^*. Mutations that disrupt the *BRCA1* C-terminal (BRCT) domain of Yph1p, the yeast Pes homolog, prevent growth indicating that the BRCT domain is critical to Pes function [10]. The *pes^vu166^* mutation truncates the Pes protein before the BRCT domain, likely resulting in a complete loss of *pes* function.

### Loss of *pes* function disrupts oligodendrocyte development

**Figure 2:**
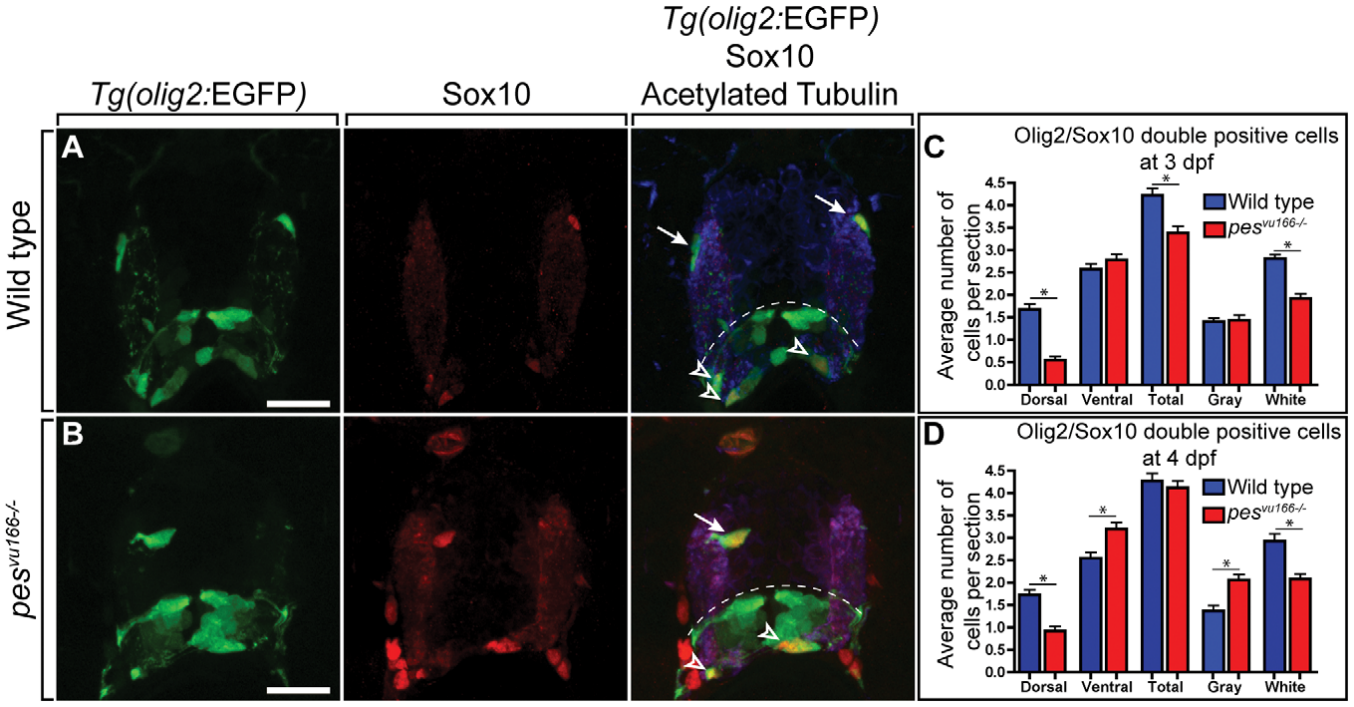
Distribution of oligodendrocyte lineage cells in wild-type and *pes^vu166^* mutant larvae. Transverse sections through spinal cords of 4 dpf wild-type (A) and *pes^vu166−/−^* (B) larvae carrying the *Tg(olig2:EGFP)* reporter and processed for Sox10 immunohistochemistry. (A) Wild type spinal cord section showing normal number and distribution of dorsal (arrows) and ventral (arrowheads) *olig2^+^*, Sox10^+^ oligodendrocytes. Dashed line marks position of pMN precursor domain. Anti-Acetylated Tubulin staining (blue) marks axon-rich white matter region of spinal cord. Dorsal *olig2^+^*, Sox10^+^ oligodendrocytes occupied white matter. (B) *pes^vu166−/−^* spinal cord section. Dorsal *olig2^+^*, Sox10^+^ oligodendrocyte (arrow) was located in gray matter region, medial to the white matter region. (C,D) Quantification of oligodendrocytes in the dorsal, ventral, gray matter, and white matter spinal cord regions at 3 and 4 dpf. Ten sections from ten larvae of each genotype were counted. Scale bar represents 30 µm. Asterisk (*) indicates p≪0.05 by Student's T-test.

The *Tg(olig2:EGFP)* reporter revealed an apparent deficit of dorsally migrated OPCs in *pes* mutant larvae. To validate this observation and more carefully investigate the distribution of OPCs, we performed immunohistochemistry to detect expression of Sox10, a specific marker of oligodendrocyte lineage cells [11], and β-Acetylated Tubulin, which labels axon tracts that mark the white matter of the spinal cord after myelination occurs (**Figure 2A,B**). At 3 dpf, the number of Sox10^+^ cells that occupied the medial, axon-poor gray matter region of the spinal cord and the ventral spinal cord was indistinguishable between *pes* mutant larvae and their wild-type siblings (**Figure 2C**). However, fewer Sox10^+^ cells occupied the dorsal spinal cord and the axon-rich white matter region in mutant larvae than in wild type (**Figure 2C**). At 4 dpf, the deficit of dorsal and white matter Sox10^+^ cells persisted in *pes* mutant larvae (**Figure 2B,D**). However, 4 dpf mutant larvae had excess ventral Sox10^+^ cells, which mostly occupied gray matter, resulting in equivalent total number of Sox10^+^ cells in wild-type and mutant larvae (**Figure 2B,D**). These data indicate that *pes* loss of function results in a transient deficit of oligodendrocyte lineage cells and disrupts their normal distribution, perhaps by disrupting OPC migration.

**Figure 3:**
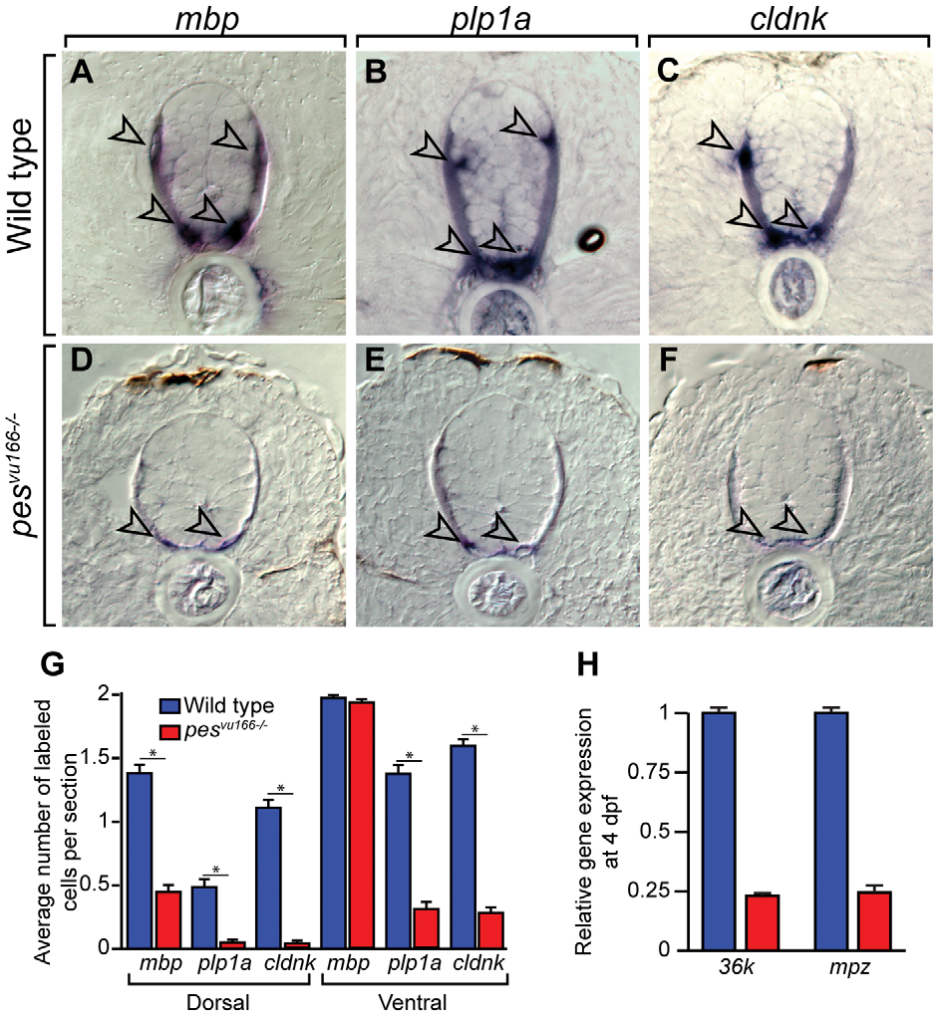
Loss of *pes* function reduces oligodendrocyte gene expression. Transverse sections through spinal cords of 4 dpf wild-type (A–C) and *pes^vu166−/−^* (D–F) larvae processed for in situ RNA hybridization. Arrowheads indicate oligodendrocytes. Reaction products are greatly reduced in mutant relative to wild type. (G) Average number of labeled cells in the dorsal and ventral spinal cord. Ten sections from ten larvae of each genotype were counted. Asterisk (*) indicates p≪0.05 by Student's T-test. (H) Relative expression levels of the CNS myelin genes *mpz* and *36k* measured by quantitative PCR.

The deficit of white matter oligodendrocyte lineage cells raised the possibility that *pes* mutant larvae have a deficit of myelination. To test this, we performed in situ RNA hybridization to detect expression of genes characteristic of differentiating oligodendrocytes. Whereas mutant and wild-type larvae had similar number of ventral *mbp^+^* cells, mutant larvae had significantly fewer dorsal *mbp^+^* cells and fewer ventral and dorsal *plp1a^+^* and *cldnk^+^* cells than wild type (**Figure 3A–G**). Additionally, the amount of reaction product detected in individual cells of mutant larvae was substantially less that in wild-type larvae for all three probes, including cells that occupied their normal positions near the pial surface (**Figure 3A–F**). Although the apparent reduction of reaction product could result from decreased probe penetration in mutant larvae, a *sox2* RNA probe detected higher levels of gene expression in mutants than in wild-type (see below). To further investigate oligodendrocyte differentiation, we performed quantitative real-time PCR to assay the relative expression levels of the CNS myelin genes *myelin protein zero (mpz)* and *36K* at 4 dpf. Both genes were expressed at levels fourfold lower in mutant larvae relative to wild type (**Figure 3H**). Therefore, *pes* function is necessary both for the proper number and distribution of oligodendrocyte lineage cells and for oligodendrocyte differentiation.

### Loss of *pes* function disrupts progression of spinal cord cells through the cell cycle

**Figure 4:**
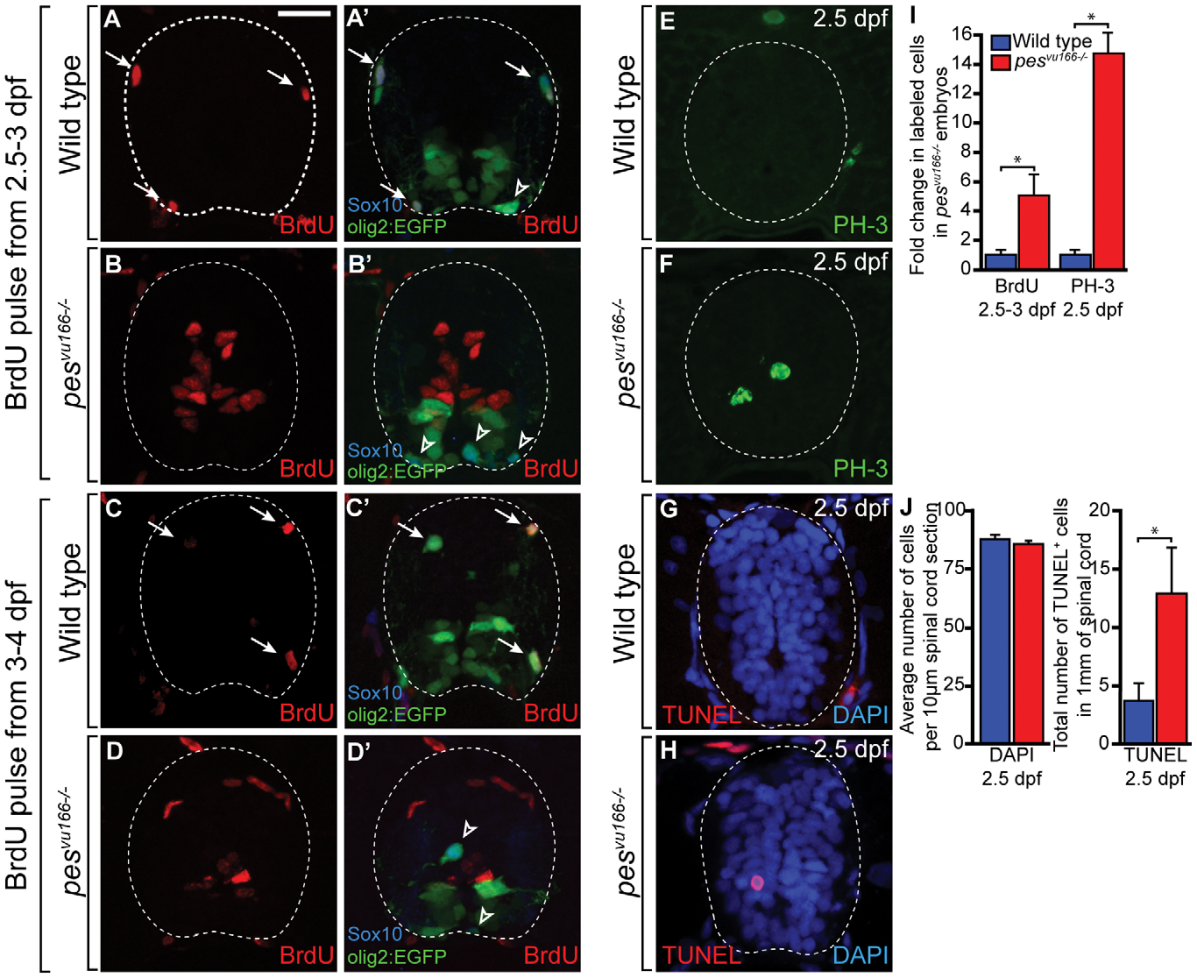
Markers of cell cycle activity are significantly increased in the spinal cords of *pes^vu166−/−^* embryos. (A–H) Transverse sections through the spinal cords of 3 dpf (A,B), 4 dpf (C,D) and 2.5 dpf (E–H) larvae processed for immunohistochemistry. (A,C) Wild type spinal cord (dashed line) section showing BrdU^+^ (arrows) and BrdU^−^ (arrowheads) *olig2^+^*, Sox10^+^ OPCs. (B,D) *pes^vu166−/−^* spinal cord sections. OPCs did not incorporate BrdU but numerous cells lining the central canal and medial septum were BrdU^+^. (E,F) Representative spinal cord sections showing the M-phase marker PH-3. (G,H) Representative spinal cord sections showing TUNEL labeling (red) to mark apoptotic cells. DAPI labeling (blue) marks cell nuclei. (I) Quantification of the change in total BrdU^+^ and PH-3^+^ cells between wild-type and *pes^vu166−/−^* spinal cords. (J) Quantification of DAPI^+^ and TUNEL^+^ cells in wild-type and mutant spinal cords. Asterisk (*) indicates p≪0.05 by Student's T-test.

The reduction in dorsal OPCs and the previously proposed role of *pes* in cell proliferation [12] raised the possibility that *pes* function is necessary for the division of OPCs after they migrate out of the pMN domain. To assay the proliferation of OPCs in mutant larvae we exposed the larvae to the thymidine analogue BrdU, an S-phase marker, at various time-points during early development. In wild-type larvae, most OPCs were labeled with BrdU when the pulses were conducted between 52 and 96 hours post fertilization (hpf), and these labeled OPCs represented the vast majority of the BrdU^+^ cells in the spinal cord (**Figure 4A,C** and data not shown). Consistent with a requirement for *pes* in cell division, there were no BrdU positive OPCs in the spinal cords of mutant larvae. However, this experiment revealed that mutant spinal cords had a five-fold increase in the total number of BrdU^+^ cells, which were mostly adjacent to the central canal and medial septum (**Figure 4B,D,I**). Additionally, the number of cells that were labeled by the M-phase marker phosphohistone H-3 (PH-3) (**Figure 4E,F**) was dramatically increased in mutants (**Figure 4I**). Nevertheless, there was no significant difference in the total number of spinal cord cells between mutant and wild type (**Figure 4J**).

One possible explanation for an increase of cell cycle activity without an accompanying increase in cell number is that cell division is balanced by cell death. To investigate this possibility we performed a terminal deoxynucleotidyl transferase dUTP nick end labeling (TUNEL) assay to label dying cells (**Figure 4G,H**). Mutant larvae had less than two-fold more TUNEL^+^ cells than wild type (**Figure 4J**), less than the five-fold increase of dividing cells. Therefore, elevated levels of cell death may not fully account for the lack of increase in total cell number despite the substantial increase in apparent dividing cells.

**Figure 5:**
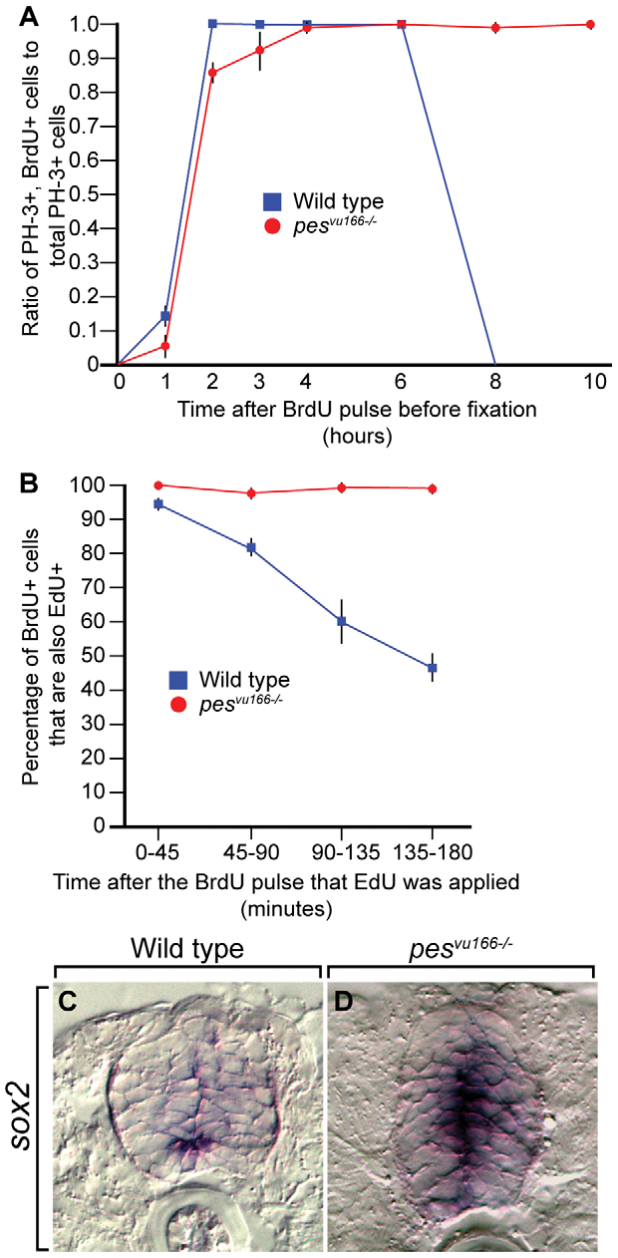
Loss of *pes* function disrupts progression of spinal cord cells through the cell cycle. (A) The time required to progress from S-phase to M-phase in the spinal cord is shown by the ratio of PH-3^+^/BrdU^+^ to total PH-3^+^ cells at the indicated time points after a BrdU pulse. (B) The length of S-phase in the spinal cord is shown by the ratio of BrdU^+^/EdU^+^ cells to total BrdU^+^ cells at the indicated time points after a BrdU pulse. Wild-type (blue) and *pes^166−/−^* (red) are shown. (C,D) Transverse sections through spinal cords of 4 dpf wild-type (C) and mutant (D) larvae processed for in situ RNA hybridization showing the expression of the neural precursor marker *sox2*.

An alternative explanation is that *pes* function is necessary for cell cycle progression. To test this idea, we investigated the cell cycle kinetics of *pes* null larvae. To determine the length of time that spinal cord cells spend in S/G2 phase, we performed a BrdU pulse-chase experiment in which larvae were labeled with BrdU and then fixed at various time-points followed by co-labeling with anti-BrdU and anti-PH3 antibodies. By finding the period of time that it took for BrdU^+^ cells to become labeled with PH-3, we calculated the time it took them to progress from S-phase to mitosis. Our results indicate that whereas spinal cord cells of wild-type larvae progressed from labeling to M phase within roughly six hours, mutant larvae had cells that were still entering mitosis as long as 10 hours after the pulse, the longest interval we collected (**Figure 5A**).

In light of this information and the known role of *pes* in regulating S-phase progression in yeast [10], we predicted that the delay in cell cycle progression in mutant zebrafish is due to a failure to progress through S-phase. To test this possibility, we conducted a second BrdU pulse-chase experiment in which we first pulsed cells with BrdU and then, at varying time-points, pulsed them with the thymidine analog EdU and fixed them immediately. Cells that co-labeled with BrdU and EdU were in S-phase during the initial pulse and had not yet exited it by the start of the second pulse. By three hours after the initial pulse, the majority of the cells in wild-type spinal cords had progressed through S-phase (**Figure 5B**). In contrast, only about four percent of the cells in mutant spinal cords had exited S-phase. Therefore, loss of *pes* function impairs the ability of spinal cord precursors to progress through S phase.

These data indicate that spinal cord precursors in *pes* mutant larvae failed to progress through the cell cycle at their normal rates, therefore, one possible explanation for the transient deficit of OPCs is that spinal cord cells are maintained as precursors at the expense of specified cell types. To test this, we examined the expression of the precursor marker *sox2*. At 4 dpf in wild-type larvae, *sox2* expression was limited to a few cells bordering the spinal cord central canal (**Figure 5C**). By contrast, many spinal cord cells bordering the central canal and medial septum of *pes* mutant larvae expressed *sox2* (**Figure 5D**), consistent with the idea that *pes* function in necessary for neural precursors to progress through the cell cycle and differentiate.

### Loss of *pes* function affects OPC and axon morphology cell-nonautonomous

**Figure 6:**
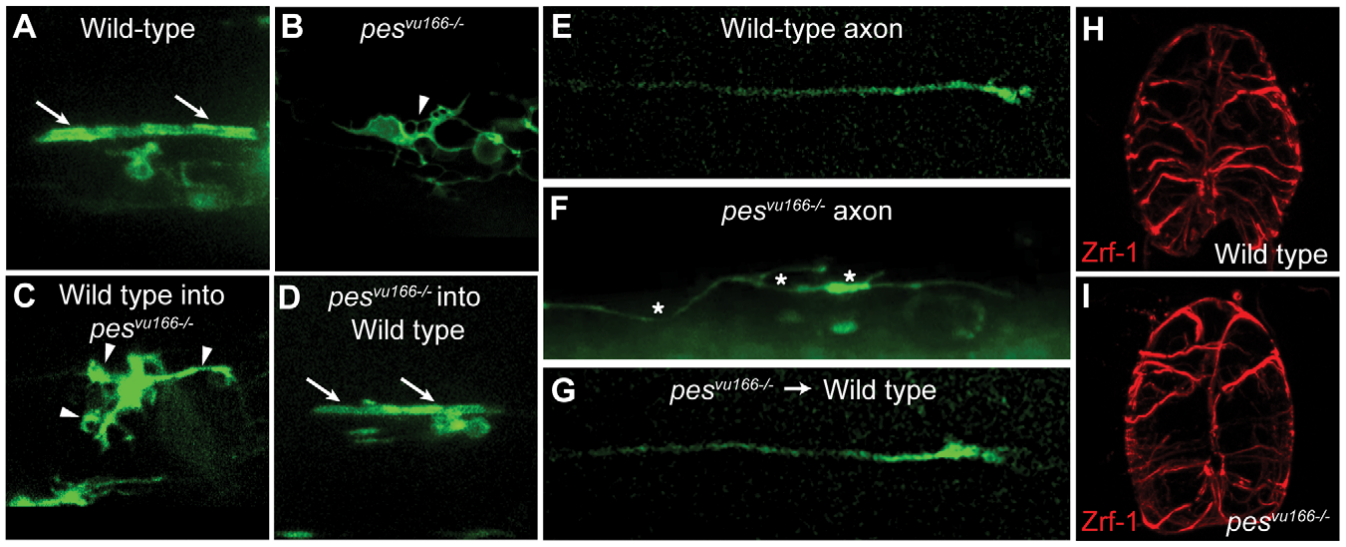
Loss of *pes^vu166^* function results in nonautonomous defects in oligodendrocyte morphology and axogenesis. (A–G) Lateral views of spinal cords in 3 dpf larvae. Expression from *Tg(nkx2.2a:mGFP)* reporter marks oligodendrocytes and axons. (A) Oligodendrocyte with normal axon wrapping morphology (arrows) in wild-type larva. (B) Oligodendrocyte with disorganized membrane morphology (arrowhead) and no evidence of axon wrapping in *pes^vu166−/−^* larva. (C) Genetically wild-type oligodendrocyte transplanted into a *pes^vu166−/−^* larva had abnormal membrane extensions and no axon wrapping. (D) Genetically mutant oligodendrocyte transplanted into a wild-type larva wrapped axons normally. (E) Axon in wild-type larva. (F) Axon in *pes^vu166−/−^* larva showing wandering trajectory, abnormal forking and growth cone collapse (asterisks *). (G) Genetically mutant axon transplanted into a wild-type larva had normal trajectory and morphology. (H,I) Transverse sections through the spinal cord of 3 dpf wild-type (H) and *pes^vu166−/−^* (I) larvae showing Zrf-1^+^ radial glia.

The abnormal distribution of OPCs in *pes* mutant larvae raised the possibility that *pes* also functions in OPC migration. To test this we performed in vivo time-lapse imaging of OPCs in wild-type and mutant larvae expressing *Tg(nxk2.2a:mEGFP)*, which encodes a membrane tethered green florescent protein under the control of *nkx2.2a* regulatory DNA. This reporter reveals the migratory behavior and process activity in the myelinating subpopulation of OPCs, as well as some axons that descend from the hindbrain through the spinal cord [3]. In wild-type larvae, OPCs extended numerous fine processes that eventually contacted and wrapped axons in tubular sheaths (**Figure 6A**). By contrast the OPCs in *pes^vu166−/−^* larvae rarely contacted axons, and instead exhibit thickened, disorganized processes (**Figure 6B**). These movies also revealed that, compared to wild type (**Figure 6E**), axons extended abnormally in mutant larvae, typified by axon wandering from the normal straight trajectories, frequent branching and repeated growth cone collapse (**Figure 6F**).

Are these defects caused by a disruption in the environment through which the cells migrate, or do they instead reflect a direct, cell autonomous requirement for *pes* in cell migration, process extension and axogenesis? To address this question we created genetic mosaic embryos containing both mutant and wild-type cells. We found that wild-type OPCs transplanted into *pes^vu166−/−^* larvae had thickened, disorganized membrane processes (**Figure 6C**), similar to those of mutant OPCs in nonmosaic larvae. Although mutant cells transplanted into wild-type larvae rarely developed as OPCs (data not shown), those that did form extended processes that contacted and wrapped axons in a wild-type fashion (**Figure 6D**). These results indicate that the cellular morphology defects of mutant larvae are cell nonautonomous. Consistent with this interpretation, *pes^vu166−/−^* neurons transplanted in wild-type larvae extended axons through the spinal cord in a manner that was indistinguishable from that of wild-type neurons (**Figure 6G**).

To gain greater insight into the cellular environment within the spinal cords of *pes^vu166−/−^* larvae, we performed immunohistochemistry using an antibody against Zrf-1, which labels the processes of radial glia in the spinal cord at 4 dpf. When compared to wild-type radial glia, we found that mutant larvae had a disorganized labeling pattern (**Figure 6H,I**), as well as regions of the spinal cord that appear to be devoid of radial glia processes. These data indicate that the spinal cords of *pes^vu166−/−^* larvae have an abnormal cellular organization, which likely impairs cell migration and axon extension.

## Discussion

### Pescadillo is required for cell cycle progression

*pes* was first identified in a zebrafish insertional mutagenesis screen on the basis of its loss-of-function phenotype, which included abnormally small eyes, brain, fins, liver and gut [8]. Subsequently, human cancer cell lines were found to express the homologous gene, *PES1*, at elevated levels [13] and *PES1* overexpression induced transformation of non-tumorogenic fibroblast cell lines [14], thus raising the possibility that Pes proteins promote cell proliferation. Consistent with this, yeast cells lacking function of *Yph1p*, the yeast *pes* homolog, underwent cell cycle arrest [10], *Pes1* mutant mouse embryos arrested as early as the eight-cell stage [12] and knockdown of *pes1* function by RNAi in two breast cancer cell lines resulted in a significant inhibition of cell growth [15].

In yeast, Yph1p, also known as Nop7p, is required for ribosome biogenesis [16], suggesting that loss of Yph1p function resulted in cell cycle arrest because of ribosome depletion. However, Yph1p can interact with many proteins that regulate cell cycle progression, particularly those that are active during S-phase [10]. In the absence of Yph1p function, yeast cells released from hydroxyurea-induced arrest proceeded through S-phase at a significantly slower rate than normal. Consistent with this result, we observed a dramatic elongation of the cell cycle, and S-phase in particular, in the spinal cords of *pes* mutant zebrafish. However, the mechanism by which Pes regulates the cell cycle has yet to be elucidated. One possibility is that Pes directly regulates cell cycle progression through its interaction with MEC1, the yeast ATM/ATR homologue, which regulates cell cycle checkpoint control [10]. Pes also interacts with CDC28, the yeast cyclin dependant kinase and a known regulator of cell cycle progression. Therefore, the failure of neural precursors to progress normally through the cell cycle in *pes* mutant zebrafish may reveal a specific role for Pes in cell cycle regulation.

### The requirement for Pes function in OPC migration and axogenesis is cell non-autonomous

Our time-lapse imaging experiments showed that OPCs had abnormal morphologies and dorsalward migration movements and that axons followed abnormal trajectories within spinal cords of *pes* null larvae. In frogs, reduction of *pes* function with antisense morpholino oligonucleotides caused craniofacial defects by interfering with the migration of neural crest cells into the brachial arches [17]. Pes interacts with insulin receptor substrate 1, which regulates insulin and insulin-like growth factor signaling [14], leading the authors to propose that Pes regulates cell intrinsic mechanisms necessary for neural crest migration. However, whether *pes* function is required in neural crest cells or the cells with which neural crest cells interact as they migrate was not tested. Our genetic mosaic experiments showed that, for OPC migration and axon extension through the spinal cord, the requirement for *pes* function is cell non-autonomous, indicating that these behaviors do not rely on cell intrinsic functions of *pes*. Both OPCs and axons require multiple extrinsic guidance cues [18], [19] expressed by specific cell populations along their trajectories. Disruption of neural cell specification and differentiation from loss of *pes* function could interfere with production of these cues, leading to defects in OPC migration and axogenesis.

### Pes function and oligodendrocyte differentiation

Knockdown of *pes* function in frogs blocked expression of retinal marker genes, suggesting that *pes* promotes cell differentiation [17]. Consistent with this, Pes can bind DNA and act as a transcription factor in reporter gene assays [20]. Our studies indicate that Pes might also promote oligodendrocyte gene expression and differentiation. Although *pes* mutant larvae had a transient deficit of oligodendrocyte lineage cells, perhaps because of an extended cell cycle, OPCs eventually formed in normal number. However, these cells expressed myelin genes at barely detectable levels by in situ RNA hybridization, a result we validated using quantitative PCR. One possible explanation is that, as with OPC migration, the failure to express myelin genes at wild-type levels is a secondary consequence of an abnormal cellular environment. In particular, because OPCs do not migrate and wrap axons normally in *pes* mutant larvae they might fail to receive signals from axons that promote oligodendrocyte differentiation. However, oligodendrocytes express myelin genes robustly in cell culture, even in the absence of axons [21]. Notably, lack of *pes* function does not cause a general block of cell differentiation because neurons appeared to express the differentiation markers acetylated Tubulin (**Figure 2**) and Hu proteins (unpublished data) normally. Taken together, these observations raise the possibility Pes functions within the oligodendrocyte lineage to promote myelin gene expression and differentiation.

## Materials and Methods

### Ethics Statement

The Institutional Animal Care and Use Committee at University of Colorado Anschutz Medical Campus approved all experiments in which animals were used. (IACUC protocol # B-85408(09)).

### Animals

Embryos were obtained by pair-wise matings of either AB, *Tg(olig2:EGFP)* [6] or *Tg(nkx2.2a:mEGFP)* [3] fish heterozygous for the *vu166* allele and kept at 28.5°C in egg water (6 g sea salt in 20 L H_2_O) or embryo medium (EM) (15 mM NaCl, 0.5 mM KCl, 1 mM CaCl_2_, 1 mM MgSO_4_, 0.15 mM KH_2_PO_4_, 0.05 mM NH_2_PO_4_, 0.7 mM NaHCO_3_), and staged according to hours post fertilization and morphological criteria [22]. For whole-mount imaging embryos were treated with 0.0002% PTC in egg water to inhibit pigment formation.

### Genetic Mapping

The *vu166* mutation was mapped to chromosome 5 using bulked segregant analysis of microsatellite markers using standard methods [7]. Whole RNA was isolated from 4 dpf mutant and wild-type larvae and converted into cDNA using a Superscript III reverse transcriptase kit (Invitrogen #18080-051). Candidate genes were sequenced using ABI 3730 DNA sequencing instruments using gene specific primers. No new sequence information was generated for submission to GenBank.

### BrdU and EdU labeling

Dechorionated embryos were labeled with 5-bromo-2′-deoxyuadine (BrdU) (Roche) by incubating them in 20 mM BrdU in EM with 10% DMSO at room temperature for the first 30 minutes. For longer incubations, the initial BrdU solution was replaced with one containing 20 mM BrdU in EM with 1% DMSO for the indicated amounts of time at 28.5°C. For co-labeling with 5-ethynyl-2′-deoxyuridine (EdU), following BrdU pulse embryos were allowed to develop at room temperature for the indicated amount of time, then incubated in 2 mM EdU (Click-iT EdU Alexafluor 555 detection kit, Invitrogen #c10338) in EM with 10% DMSO for 30 minutes at room temperature. The fish were then fixed in 4% paraformaldehyde (PFA) in PBS with 116 mM sucrose and 150 µM CaCl_2_ at 4°C overnight.

### Immunohistochemistry and EdU detection

Fixed embryos were imbedded in 1.5% Agar with 5% sucrose and transferred to a 30% sucrose solution in scintillation vials and incubated at 4°C overnight. The blocks were then frozen and cut into 10 µm transverse section on a cryostat microtome. The sections were incubated with rabbit anti-Sox10 (1∶1,000) [9], mouse anti-β-acetylated tubulin (1∶1000, Sigma), rabbit anti-phosphohistone H-3 (1∶1,000, Upstate Biotechnology) and anti-BrdU (1∶100, Developmental Studies Hybridoma Bank) antibodies. For fluorescent detection of antibody labeling we used Alexafluor 568 and Alexafluor 647 goat anti-mouse and goat anti-rabbit secondary antibodies (1∶200, Invitrogen). To detect EdU incorporation, we incubated the slides in 250 µL of the EdU Detection Reaction mix (prepared according to the manufacturer's direction) for 40 minutes at room temperature. Images were collected on a Zeiss Axio Observer 200 microscope with a PerkinElmer Ultraview Vox spinning disk confocal unit equipped with Volocity imaging software (PerkinElmer). Images were contrast enhanced using either Volocity or Photoshop (Adobe CS4).

### RNA in situ hybridization

Embryos were fixed in 4% PFA in DEPC treated PBS overnight at 4°C and stored in methanol at −20°C. Anti-sense RNA probes were synthesized from a linearized plasmid DNA template using Roche digoxygenin labeling reagents and either T3 or T7 RNA polymerases (New England Biolabs). The in situ hybridization was performed using the standard protocol [23]. Images were obtained on a Zeiss Axio Observer 200 microscope with Volocity imaging software. Images were contrast enhanced using Photoshop (Adobe CS4).

### Quantitative PCR analysis

Total RNA was isolated from 4 dpf mutant and wild-type larvae and converted to cDNA as above. Relative gene expression was determined using TaqMan reagents (Applied Biosystems #4304437) according to the manufacture's instructions. Probes were used against zebrafish *mpz* (Assay ID Dr03131917_m1) and *36K* (Assay ID Dr03438575_m1) using *Rpl13a* (Assay ID Dr03101115_g1) as an internal control.

### Chimeric analysis of zebrafish embryos

We used the *Tg(nkx2.2a:mEGFP)* line for chimeric analysis by cell transplantation. 30–50 cells from donors at the ‘high’ stage of blastula development obtained by crossing *pes^vu166+/−^*; *Tg(nkx2.2a:mEGFP)* or *Tg(nkx2.2a:mEGFP)* wild-type adults were transplanted into same-stage wild-type hosts or hosts derived from *pes^vu166+/−^* adults respectively. Host embryos were raised at 28.5°C and imaged at 3 dpf. Donor embryos were immediately genotyped by PCR. Larvae used for imaging were anesthetized using 3-aminobenzoic acid ethyl ester, and mounted in a 2% agarose lateral imaging mold [24]. Images were captured using a 20× dry (NA = 0.75) objective mounted on a motorized Zeiss Axiovert 200 microscope equipped with a PerkinElmer Ultraview Vox spinning disk confocal unit and heated stage to maintain embryos at 28.5°C. *Z* image stacks were collected and three-dimensional data sets were created using Volocity imaging software. Images were deconvolved followed by contrast enhancement using Volocity.
